# Treatise on an alternative perspective on the two-process model of sleep regulation

**DOI:** 10.1038/s44323-025-00038-0

**Published:** 2025-06-18

**Authors:** Frederik Bes

**Affiliations:** 1 Clinic for Sleep & Chronomedicine, St. Hedwig-Krankenhaus, Berlin, Germany; 2 Institute of Physiology, Charité Universitätsmedizin Berlin, Berlin, Germany

**Keywords:** Circadian rhythms and sleep, Diseases of the nervous system

## Abstract

The two-process model of sleep regulation, groundbreaking for the field of sleep research, describes sleep-wake behavior as regulated by the interaction of a homeostatic sleep-wake drive, process S (‘sleep debt’), and a circadian pacemaker process C, that “gates” S between two thresholds. The model has achieved great acceptance and application. The conception of the model would have been fundamentally different if one of the model’s creators (Borbély) had retained an alternative conceptual perspective, which he initially described at the time but did not elaborate on. The alternative considers the interaction of two sleep-wake processes more or less equivalent in strength and would a) allow the integration of REM sleep as an important circadian model constituent, b) adequately model four major aspects of the 24-h time course of sleep propensity, c) address some unresolved problems with the current model, and d) not conflict with the vast majority of published experimental research.

## Introduction

It has now been about forty years since the two-process model of sleep regulation, groundbreaking for the field of sleep research, was proposed^1,2^. This model describes sleep-wake behavior as regulated by the interaction of a homeostatic sleep-wake drive, process S (‘sleep debt’, rising during wakefulness and declining during sleep), and a circadian pacemaker process C, that “gates” S between two thresholds. The publication of this deterministic conceptual model has greatly stimulated fundamental research and undisputedly has contributed much to the present understanding of human sleep. Although many successor models have been proposed, often extending the original with necessary additional components (for a review, see ref. ^3^), the model in essence has remained conceptually unchanged, that is a threshold-based interaction between a homeostatic sleep propensity and a circadian wake propensity process^4–6^.

This paper attempts to emphasize that the conception of the model would have been fundamentally different if one of the creators of the model (Borbély) had retained an alternative conceptual perspective on the two constituent processes, which he initially described at the time but did not elaborate on. Instead of a homeostatic sleep process gated by a circadian alerting signal, the alternative perspective considers the interaction of two sleep-wake processes that are more or less equivalent in strength. Borbély did not elaborate on that initial alternative, because he pursued a different track (focused on modeling duration and, in collaboration with Daan and Beersma^2^, also the timing of sleep), not because it would be inherently false. Furthermore, the treatise attempts to demonstrate with several crucial examples that retaining the initial alternative perspective, while adopting a multiplicative interaction between the two processes might even enhance the explanatory capacity of the two-process model. Thus, the alternative perspective would a) allow the integration of REM sleep as an important circadian model constituent, b) be able to adequately model four major aspects of the time course of sleep propensity over 24 h, c) be capable of addressing some unresolved problems with the current model, and d) not be in conflict with the vast majority of experimental research that has been published within the framework of the current two-process model.

## Theoretical considerations

### Two interacting processes as a conceptual model

Borbély’s original approach in 1982 was based on the assumption that sleep propensity (as well as sleep duration) is determined by the combined action of two separate processes. One, Process S, is an exponential function of sleep and wakefulness, increasing during wakefulness, and decreasing during sleep, while the other, Process C, is a sleep process controlled by a circadian oscillator and unaffected by the occurrence of sleep and wakefulness. The combination (i.e., summation^1^, ^p.199, line 11^) of S and C was indicated to correspond to total sleep propensity. Interestingly and noteworthy, Borbély introduced the variable $\bar{{\rm{C}}}$, being the inverse, or mirror image, of Process C, and “reflecting the circadian variation of the sleep threshold which is highest when circadian sleep propensity is lowest (i.e. in the afternoon)”^1^, ^p.199^. This shows that he preferred the deterministic principle of ‘something reaching a threshold’, with the implicit consequence of a state change. Accordingly, total sleep propensity is represented by the difference between S and $\bar{{\rm{C}}}4$^4^, and when it reaches zero, this causes a state change in the model, from sleeping to waking. Remarkably, in the same publication Borbély extended the model to also consider REM sleep. He supposed that REM sleep propensity (R) could be represented by a horizontal line, indicating a constant level during the regular sleep-wake cycle, because it is not influenced by sleep or waking. He then defined REM sleep propensity as the difference between R and $\bar{{\rm{C}}}$^1^, ^p.200^.

In 1984, Daan and Beersma, who were working on a similar concept, decided with Borbély to publish a joint paper on the two-process model, to predict the timing of human sleep for a variety of different sleep-wake schedules^2^. The modifying contribution of Daan and Beersma is evident from their earlier work, reported in a book chapter that should have been published in 1982 but was delayed until 1984^7^. Their approach used a second threshold to describe the transition from waking to sleeping, and this was incorporated in the model presented in the joint paper. Thus, sleep onset is triggered when S reaches the upper threshold H, awakening occurs when S reaches the lower threshold L. The circadian variation of both thresholds are controlled by a single pacemaker. The frequency of sleep-wake alternations depends on the distance between the two thresholds and on the rate of breakdown and buildup of Process S. It was assumed that external conditions could influence the threshold levels. For example, sleep deprivation could be simulated by increasing H, bed rest, darkness, or absence of social stimuli by decreasing H, and “…the sounding of an alarm clock could effectively equate to a sudden increase in L”^2^, ^p.R163^. A small but noteworthy detail is that although the word “process C” appears only once in the entire joint article, it has ever since become common to use the designation “process C” to refer to the circadian threshold process (i.e., the process originally denoted by Borbély in 1982 as $\bar{{\rm{C}}}$). Regarding REM sleep, the authors of the joint paper simply referred to Borbély’s 1982 publication, noting that “…when extending the model to incorporate REM sleep, we will have to examine whether the present version of the model is sufficient to account for the available data”^2^, ^p.R174^ and left it at that. Thus, REM sleep has no specific meaning or place in the two-process model.

Apart from the nature of the interaction (see below), the two-process model remained essentially unchanged in the decades that followed, i.e. a threshold-based interaction between a homeostatic sleep propensity and a circadian wake propensity process^4–6^. The literature citing the model increasingly emphasized the mutually *opposing* nature of the two processes. For example, Landolt writes, referring Franken & Dijk^8^: “In humans, we think today that the circadian system opposes homeostatic changes in sleep pressure, to enable healthy people to stay awake and alert throughout a normal waking day despite accumulating sleep pressure associated with wakefulness”^9^, ^p.85&86^.

### The initial linear nature of the proposed interaction is questionable

Over the four decades that the two-process model exists, considerable progress has been made in investigating and describing the interaction between homeostatic and circadian processes, not least through the use of powerful experimental protocols such as forced desynchrony (FD), which can separate endogenous circadian from evoked components of daily rhythms in multiple parameters^10,11^, or ultra-short sleep-wake schedules^12–14^. But apparently, little progress has been made in verifying the threshold character of the interaction as described in the model.

**Fig. 1:**
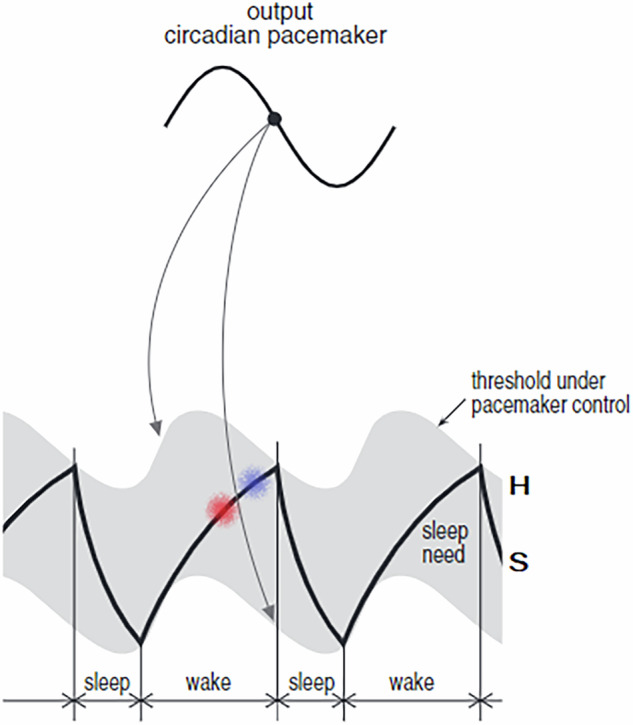
Sleepiness in the two-process-model. The difference between S and H (the upper threshold of process C) would correspond to the amount of sleepiness at any point in time: the more S approaches H, the sleepier the subject. But this linear kind of interaction cannot explain why one would tend to fall asleep already in the afternoon, about where the red, lower cloud is placed (the afternoon nap-zone), and why one shortly thereafter, without having slept, would turn again less sleepy in the early evening at the wake maintenance zone, about where the blue, upper cloud is placed. On the contrary, linear interaction would predict lower sleepiness in the afternoon and higher sleepiness in the early evening, which is at odds with results abundantly reported in the literature. (Figure adapted from^82^; copyright © 2005, by permission of Sage Publications).

The propensity for sleep or wake was assumed to depend upon the distance of S to the upper resp. lower thresholds of C^4^. Thus, the gating interaction of S and C was originally considered linear, allowing only additive or subtractive operations^15^. However, a linear relationship between two 24-h based oscillators cannot easily explain ultradian fluctuations in sleep propensity. It demonstrably is at odds with two well-described components of the sleep-wake cycle: the afternoon napping zone, ANZ, and the evening wake maintenance zone, WMZ (see Fig. 1).

Evidence for the WMZ stems from early observations of a reduced sleep propensity near bedtime^16–18^. These findings were confirmed in a series of studies with ultra-short sleep-wake schedules, reporting that sleep propensity reaches a circadian minimum a few hours before the habitual night-sleep^12,19^, and by studies using FD^20,21^.

Evidence for the ANZ stems from many studies using very different protocols: “free running” sleep-wake cycles^22^; constant darkness over 72 hours^23^; constant routine^24^; ultra-short sleep-wake cycles^12,24–26^; Multiple Sleep Latency Tests (MSLT)^27–29^; field surveys on napping^30–32^; unintentional sleep episodes^33^; performance- and vigilance tests^34–36^; traffic accidents^37,38^; and “big data”, using aggregated mobile phone calling patterns of a very large number of individuals^39^.

Although Borbély and colleagues reported that they were able to simulate the “daytime trough in sleep latency” with the two-process model^40^, they could only do so with additional assumptions about the initial conditions, such as different shapes (amplitude, skewness) of the two threshold processes, with H being a pure sine and L a skewed sine function, as well as taking into account that as a consequence, subsequent evening sleep had to be initiated without S reaching the altered gradient of threshold H. The latter was explained by “The S curve (….) does not attain the upper threshold H at sleep onset, since under everyday life conditions bedtime is determined by clock time, personal habits, social conventions, anticipated wake-up time, and so on, rather than exclusively by a physiological process.”^40^, ^p.40/152^ Adding noise to the threshold could possibly explain occasional naps, but the inherent randomness of an added stochastic variable precludes an explanation for the fact that naps occur preferentially around the time of the ANZ and less frequently around the time of the WMZ. In any case, the two-process model with the originally supposed linear interaction of the two constituent processes has difficulties in adequately describing naps during the day. The occurrence of multiple naps like within the MSLT protocol can only be explained when the distance between the upper and lower threshold of process C is decreased, but this is not unproblematic because the two-process model does not contain a variable that compares the levels of the thresholds. The individual levels of the thresholds are pre-assumed by the simulator, they are set as initial condition at every single test run. Lowering H at some time during one and the same test run would be comparable to the action of a deus-ex-machina.

### A non-additive component was included in the interaction

In the mid-1990s, interpreting FD studies, Dijk and Czeisler argued that under usual conditions (entrainment) the phase relationship between sleep-wake cycle and circadian pacemaker apparently can promote the consolidation of nocturnal sleep and daytime wakefulness^20^. Subsequently they began to label the interaction between S and C, somewhat ambiguously, as “in part non-additive” but, notably, without giving further details about what non-additive in this case could mean^21^. Thus, the authors attributed the existence of the WMZ to a consolidation of daytime wakefulness by a putative circadian drive for wakefulness, that gradually becomes stronger on the rising limb of the endogenous rhythm of core body temperature, reaching its maximum in the early evening, close to the rise of melatonin concentration and thereafter declines again^41,42^. Nevertheless, the phenomenon of the ANZ, which usually occurs not long before the WMZ and which seems therefore difficult to reconcile with this gradually increasing wake signal, was ignored. Remarkably, this “putative circadian alerting signal”^42^ was not generally included in operating diagrams (i.e., those generally depicted to see all the essentials of the model at a glance) of the two-process model, nor has its relationship with the thresholds been discussed or explained^4–6^. Nevertheless the ‘consolidation’ hypothesis seems to be widely accepted today, apparently as an independent fact, although it remains unclear where the underlying circadian alerting signal fits into the model and whether it could be synonymous with the lower threshold of C, or even with C itself.

### Sleep restriction appeared to mask the influence of C

In 2012, a group of researchers reported results of a forced desynchrony study, thus separating homeostatic and circadian influences on sleep, while comparing normal and elevated homeostatic pressure. The latter was achieved under conditions of severe sleep restriction (14 participants having a 28 h rest-activity schedule, with 1:5 sleep-wake ratio [4.7 h sleep, 23.3 h wake], compared to 13 participants having a habitual 1:2 sleep-wake ratio [9.3 h sleep, 18.7 h wake]^43,44^. The data confirmed that with a 1:2 sleep-wake ratio, the effect of the circadian process on sleep propensity (operationalized by sleep efficiency) is pronounced, including a WMZ at a phase equivalent to evening time for a normally entrained individual. But when the homeostatic drive was experimentally elevated by sleep restriction (sleep-wake ratio 1:5), the circadian effect appeared to be masked. Sleep propensity was very high at all circadian phases and no WMZ could be observed. Of note, fluctuations in core body temperature were not different under either condition, indicating comparable endogenous circadian cycles and hence comparable fluctuations of Process C^43^. The authors concluded that if the homeostatic pressure for sleep is sufficiently high, then the circadian drive for wakefulness is masked or can be overridden. In addition, they concluded^44^ “(…) that the two-process model for sleep regulation may best describe the (…) interactive effects of the homeostatic and circadian processes (…) in the absence of sleep restriction”^44^, ^p.944^ and suggested that “(…) this model may be most applicable to non-restricted sleep opportunities.”^44^, ^p.948^ If, however, a model of sleep regulation does not allow representation of the interaction of its main constituents under conditions of severe sleep restriction, this would indicate an unexpected limitation of the scope of application of the model^45^.

### A consideration to retain an original perspective

The above shows that the threshold principle, as attractive as it is, entails some limiting aspects that have not been adequately resolved so far. Apart from the fact that it is still largely unknown how the thresholds are physiologically enacted, the conceptual perspective of deterministic gating via thresholds apparently impedes a clear view on the interaction between S and C. Initially, the interaction was considered linear (additive) but in forced desynchrony studies evidence was found for partly non-linear interaction^21^ and until today the exact nature and theoretical framework of that interaction is unclear^6^. In a science-philosophical way, the conceptual deterministic approach does not seem to work quite well in this case and it could be interesting to consider whether another conceptual approach might be more appropriate. Sleep is a complex phenomenon, mediated by numerous changes in internal, behavioral and environmental factors associated with the sleep–wake cycle. This multifactorial complexity argues for a probabilistic perspective rather than a deterministic one. Viewed in this way, human sleep propensity can be defined as likelihood of falling asleep or staying asleep at a given time.

When we go back to the very beginning of the two-process model, Borbély introduced a homeostatic and a circadian sleep process that, in combination, represent the total sleep propensity. This is obvious from the following citation: “In the model, Process S is regarded as the major sleep-dependent component of total sleep propensity. The latter is assumed to be the combined result of Process S and of a circadian sleep process (Process C). The level of Process C corresponds therefore to the circadian component of sleep propensity.”^1^, ^p.199^ So it must be noted that initially, both processes were actually considered *sleep* processes. At the same time, however, Borbély began to focus on the threshold nature of the circadian process, because he was interested in modelling timing and duration of sleep. For this reason he introduced the variable $\bar{{\rm{C}}}$, which is the mirror image of the circadian sleep drive and in fact represents the circadian drive for wakefulness. At first glance this may seem merely philosophical (while mathematically equivalent), but it is a crucial conceptual step, because it means that he no longer regarded sleep propensity as determined by two drives working in the same direction (sleep), but by two drives that are opposite to each other, sleep (homeostatic) versus wake (circadian gating). With this move, he conceptually reduced circadian sleep to merely a consequence of low circadian drive for wakefulness. But most importantly, in doing so, he eliminated the possibility of considering all the potentially relevant intrinsic properties of circadian sleep as an essential part of the model.

A very interesting question, then, is what the possible consequences would be if we instead continued to focus on the circadian process as a *sleep* process, thus retaining the conceptual perspective that Borbély initially considered but never elaborated on, and thus following a track that is rather focused on sleep propensity.

### Possible consequences of retaining Borbély’s original perspective

The first consequence would be that we then retain the attractive modality of simply combining the needs for two fundamentally different types of sleep to derive total sleep propensity. In doing so, if we adopt a probabilistic perspective and consider the homeostatic and circadian sleep drives as a reflection, each within its own quality, of likelihood of falling asleep at a given time, we obtain a logical argument for being allowed to multiply their values, and thus for a continuous, non-linear relationship: P(total sleep propensity) = P(homeostatic sleep propensity).P(circadian sleep propensity). Thus, the probabilistic approach certainly does not lead inexorably to a multiplicative interaction, but that approach does offer an attractive opportunity to consider multiplication as a valid argument for a non-linear interaction.

A second, major consequence would be that with two fundamentally different types of sleep, homeostatic and circadian, the opportunity arises to imply REM sleep as key circadian element in the model. Quality and intensity of human consciousness varies greatly during the sleep-wake cycle and depends on the brain’s three physiologically identifiable behavioral states: Wakefulness, NREM- and REM sleep^46–49^. Therefore, if REM sleep is recognized as one of the ‘three primary states of being’^50^, then it follows that a conceptual model that attempts to describe sleep-wake regulation, and includes the contribution of REM sleep, can be considered more comprehensive. It has been long known that the drive for REM sleep contains the essential attributes of a circadian process^14,21,51–56^. The question here is how to quantify this drive. Referring this, Borbély and Achermann stated that “One of the difficulties in modeling REM sleep regulation is that in contrast to non-REM sleep, there is no obvious marker of REM sleep intensity. If an intensity dimension of REM sleep is indeed not existent, then a rise in REM sleep pressure must manifest itself exclusively in an increased duration of REM sleep.”^57^, ^p.565^ We must disagree with the latter statement, firstly, because the FD protocols have clearly shown that REM duration is in part dependent on disinhibition by process S^21^. But secondly, increased REM sleep pressure is more likely to manifest itself in shortened REM latencies, not necessarily in increased REM sleep durations. In full analogy to how sleep pressure can be quantified from sleep latency, a premise that is the raison d'être of the MSLT, REM pressure can be quantified from REM latency.

### Borbély’s original concept as a starting point for an alternative interpretation

Thus, not following Borbély’s decision (to focus on thresholds) opens up the interesting possibility of an attractive alternative interpretation of the 2-process model that conceptually is differently balanced than the currently prevailing one, combining two fundamentally different types of sleep to infer sleep propensity, while recognizing REM-sleep, alongside slow-wave sleep, as an important constituent.

In terms of effect, the two different processes in the alternative interpretation can be assumed to be of equivalent strength, implying equal contribution to sleep consolidation under normal entrained conditions, consistent with the arguments of Dijk and Czeisler^20^. Furthermore, they can be characterized as circadian and homeostatic sleep-wake propensities that, by virtue of their multiplicative continuous interaction, sometimes work in opposition and sometimes in synergy to represent total sleep propensity at a given time. This makes thresholds in the model redundant.

What would be the model features and eventual further merits of this alternative interpretation? The following provides a summary of the little that has been published on the subject, supplemented where appropriate with possible further data or theoretical considerations and insights.

### The time course of sleep propensity over 24 h under usual (entrained) conditions

It has previously been shown that diurnal fluctuations in sleep propensity such as napping and the WMZ can be adequately modeled by assuming a multiplicative interaction between two sleep drives^58,59^, although so far not with the explicit theoretical embedding in the two-processes model as given here. From here on, for reasons of convenience, this alternative interpretation of the 2-process model will be referred to as the “S.R model”, in which the original homeostatic component S remains as it is and C is replaced by R because in compliance with what has been argued above, the circadian component is constrained to an actual sleep process, represented by REM sleep propensity and quantified from REM-latency^55^, and both components, S and R, are multiplied^59^. To distinguish, the classical 2-process model will be referred to as the “S-C model”.

**Fig. 2:**
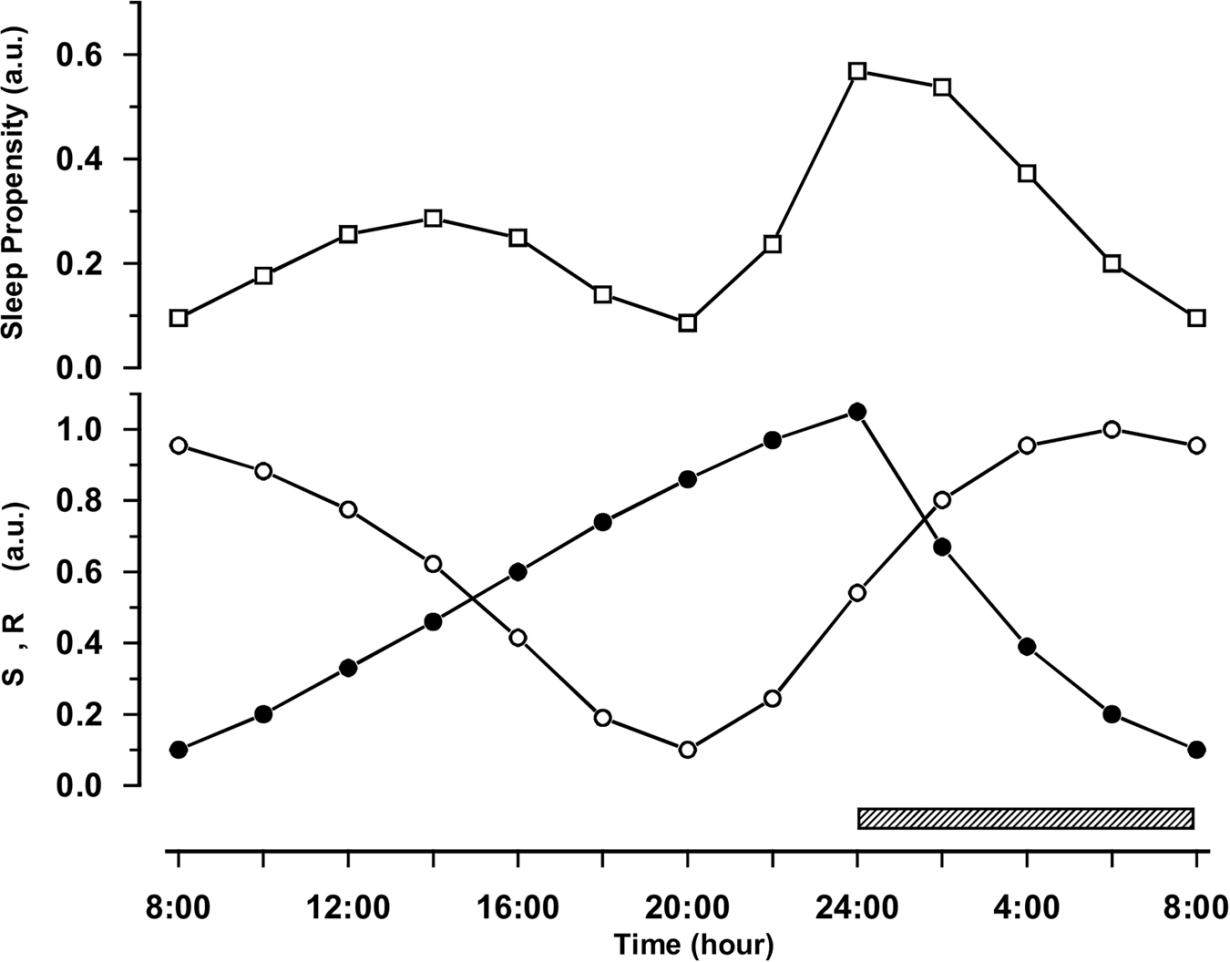
Sleep propensity over 24 h estimated with the S.R model under normal, entrained conditions. The time courses for the homeostatic sleep drive S (filled circles) and the circadian sleep drive R (open circles) are depicted in the lower panel. The scale is relative and runs in arbitrary units (a.u.) from 0 (low) to 1 (high), although S and R are set so that they never actually reach zero (discussed in ref. ^59^). The sleep propensity function (open squares, upper panel) was computed by multiplying the values of S and R at each point in time. An eight-hour sleep episode (hatched bar) is assumed to take place between 24:00 h and 08:00 h (adapted from ref. ^59^; copy right 2009, by permission of Oxford University Press).

Figure 2 shows the estimated time course with the S.R model, of sleep propensity over 24 h under entrained conditions, so, with a usual phase (sleep onset at midnight) and duration (1:2 sleep-wake ratio) of nocturnal sleep. The parameters for S and R (lower panel) were estimated from a nap study (for details, see refs. ^55,59^). The sleep propensity function (upper panel) shows four characteristics, (a) a primary, major peak at nighttime, (b) a secondary, smaller peak in the afternoon (the ANZ), (c) a first local minimum in the morning, and (d) a second local minimum in the early evening hours (the WMZ).

**Fig. 3:**
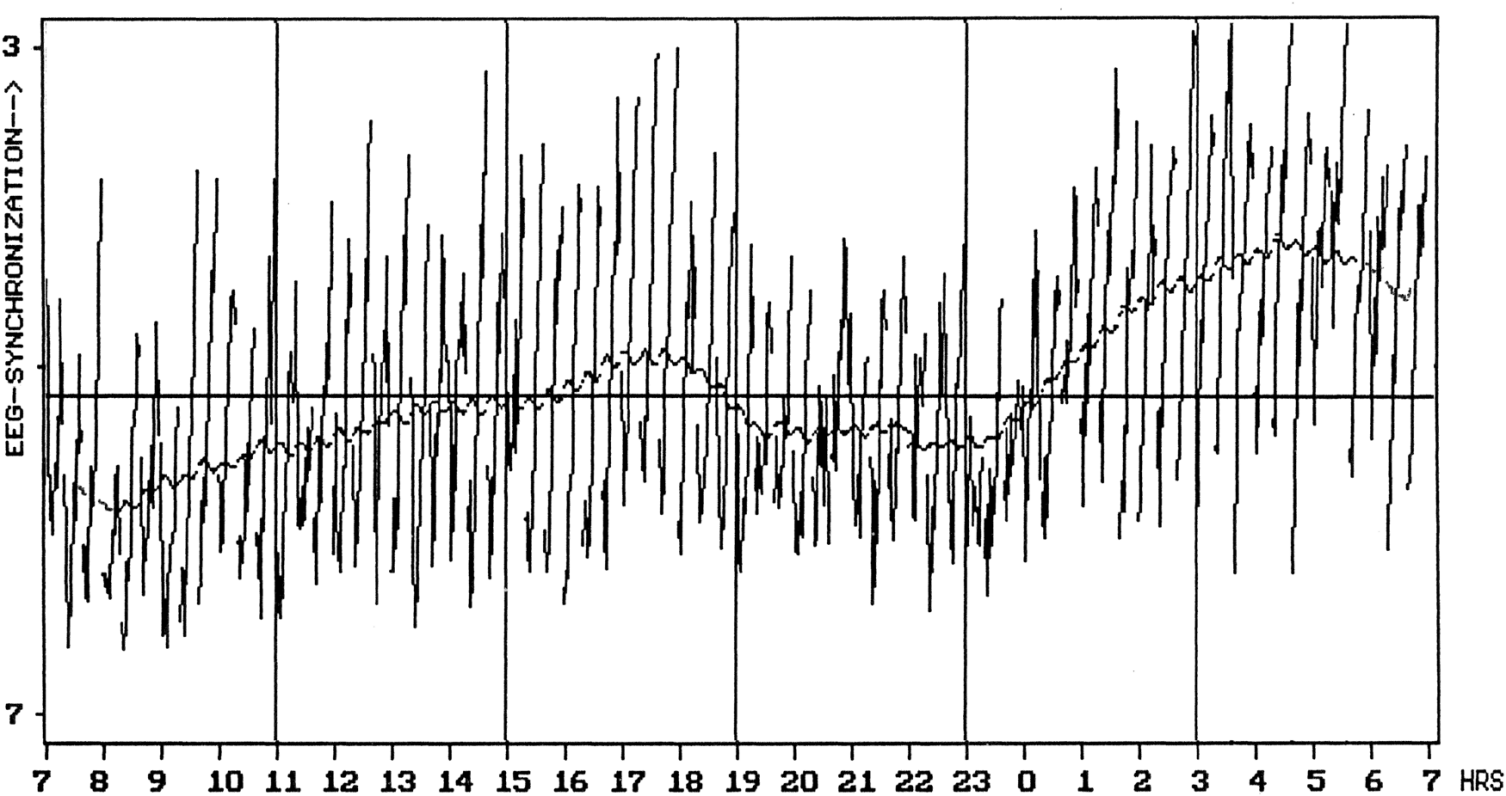
Measured sleep propensity over 24 h (local clock time). The depicted 72 steeply rising lines represent the degree of EEG-synchronization (frequency slowing of the EEG, in arbitrary units, for every 30 s while falling asleep) in each of the 72 7-min naps, averaged across all 14 participants. The smoothed line is a 2-h moving average over the 72 nap lines and displays the 24-h time course of sleep propensity, with visually prominent daily variation. Clearly visible are two peaks, the larger one representing the elevated sleep propensity that is usual at night, and the secondary, smaller one in the late afternoon that likely represents the afternoon napping zone. Also visible are two minima, one in the morning around the habitual time of getting up, and a second one in the evening that likely represents the wake-maintenance zone (scaling inverted, adapted from ref. ^60^).

The estimated time course appears to match well with actual empirical data (Fig. 3). The latter was inferred from a collaboration with Lavie and colleagues using an ultrashort sleep-wake protocol over 24 h (7/13 min sleep/wake), in which 14 healthy male participants were recorded polysomnographically^60^. They participated in a study on the influence of exposure to bright or dim light in the evening. The propensity to fall asleep was quantified by the amount of EEG synchronization per 30 s^61^, on a scale of arbitrary units running from 0 (maximum synchronization) to 10 (maximum desynchronization), and actually shows the slowing of the EEG while falling asleep in a nap.

Figure 3 shows the results from the baseline (dim light) group. The main features of the experimentally determined time course of sleep propensity are qualitatively in excellent agreement with the time course according to the S.R model depicted in Fig. 2. The only difference, of several hours in phasing of the ANZ in the estimated and experimentally determined time course, could be due to cultural and/or geographical differences, explained by the fact that the estimation was based on data from participants from Berlin, Germany, while the experimentally determined data were from participants in a Mediterranean environment (Haifa, Israel).

The straightforward result of multiplying R by S therefore appears to describe the four main characteristics of the 24-h time course of sleep propensity very well. It should be underlined that additional assumptions, as in the S-C model for the shape or time course of the thresholds to describe the phenomena of ANZ and WMZ, are not necessary.

### Effects of a daytime nap

**Fig. 4:**
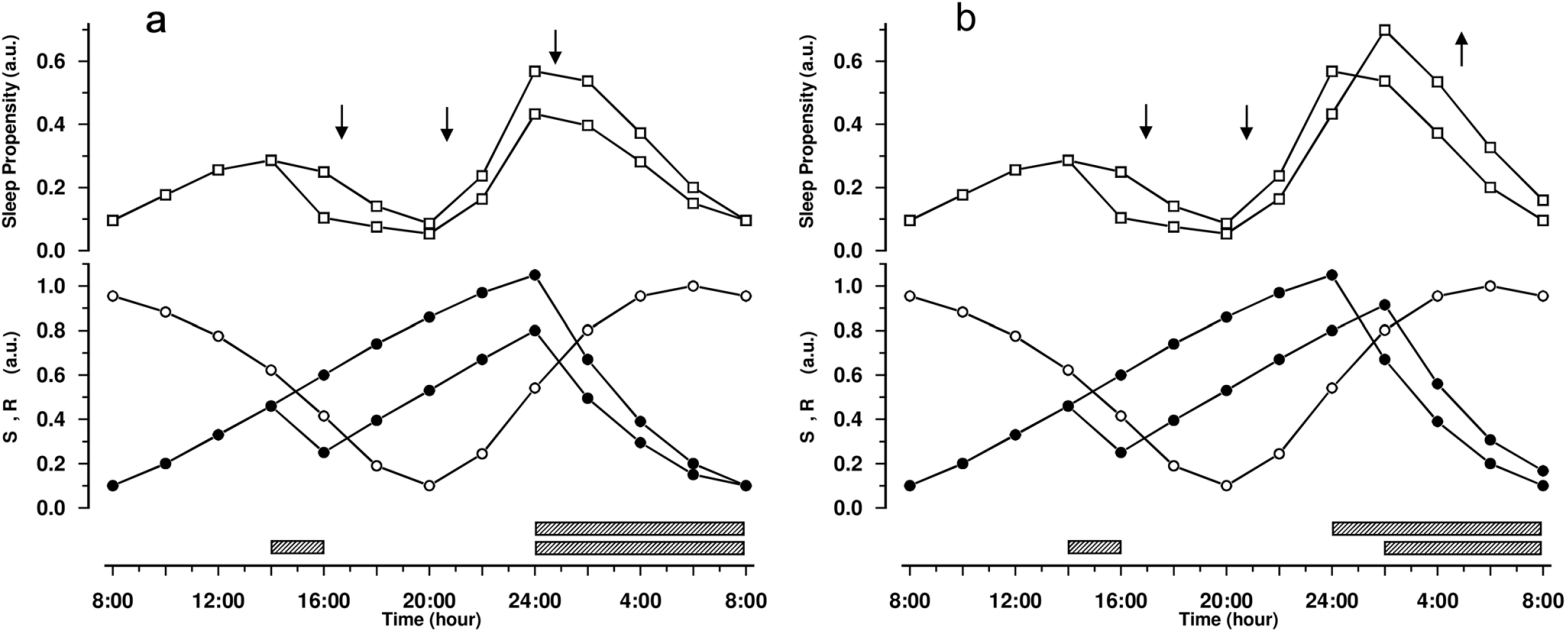
Effects of a daytime nap, under usual, entrained conditions. A 2-h nap initiated at 14:00 impacts sleep propensity differently in the subsequent night, dependent on when the subject falls asleep at night. S (filled circles) and R (open circles) are represented in the lower panels, the resulting sleep propensity function (open squares) in the upper panels. The hatched bars indicate sleep episodes. The directions of the shift in the sleep propensity function (compared to the initial situation without a nap) are indicated by arrows. **a** After the daytime nap, the subject falls asleep at his “usual” bed time at midnight; the sleep propensity function indicates lower than usual values across the night which may be indicative for shallow sleep. **b** After the daytime nap, the subject is unable to fall asleep at his “usual” bed time at midnight; the increased sleep latency reduces nocturnal sleep time, and causes further accumulation of S that, in combination with increasing levels of R, imply increasing levels of sleep propensity until the usual levels for sleep onset are reached (or exceeded).

Figure 4 shows the modeled effects of a two-hour afternoon nap on the subsequent course of sleep propensity under usual, entrained conditions. Visualizing a quantification that can be verified experimentally in detail, the S.R model estimates a time course for post-nap sleep propensity that is roughly parallel to the time course without napping, but at a lower level. When attempting to fall asleep at night after the nap, the effect on sleep propensity can fluctuate between two extremes depending on when the subject manages to fall asleep. If the subject actually manages to fall asleep immediately (Fig. 4, left pane), the low levels of the nocturnal sleep propensity suggest shallow sleep, which in turn may indicate low sleep efficiency and quality. Alternatively, the subject’s sleep onset is delayed and he or she may not fall asleep until sleep propensity has reached or exceeded the usual levels for sleep onset (Fig. 4, right pane), thus reducing the time spent asleep. Several studies indeed confirmed that napping is related to worse sleep quality^62^, reduced sleep efficiency^63–65^ and sleep duration^64–66^, and increases in sleep onset latency^67^.

### Effects on the afternoon napping zone when sleep is advanced or delayed

Looking closely at the time courses of sleep propensity and the underlying processes S and R under usual entrained conditions (Fig. 2) and taking into account the relatively long-lasting effect of just one daytime nap on subsequent sleep propensity (Fig. 4), suggests that a phenomenon such as the ANZ might depend on the timing of previous sleep periods. Indeed, simulations in which the main sleep period was delayed or advanced have demonstrated that the extent of the ANZ is variable, changing its magnitude and phase in response to the phase of the preceding main sleep period (for graphical details, see ref. ^59^, ^p.395^). With main sleep period delays of up to 4 h, the subsequent ANZ decreases in magnitude, and finally disappears completely with shifts larger than 6 h. As an aside, this could explain why Dijk and Czeisler found no ANZ in their FD experiments, at least in those in which subjects were scheduled to a 28 h rest-activity cycle^21^. The resulting consistent 4-h phase delays of the main sleep period in that protocol practically precluded occurrence of the ANZ.

Stepwise advancing the main sleep period causes the subsequent ANZ to increase in magnitude and to advance into the morning hours. Interestingly, with an advancement of the main sleep period from 2 to 4 h, the primary and secondary peaks in sleep propensity are of comparable magnitude, and sleep propensity over 24 h then exhibits an essential bimodal distribution.

For a comprehensive discussion of supporting empirical studies on effects on delaying or advancing sleep times, the reader is referred to Bes et al.^59^.

### Effects on the wake maintenance zone

Simulations with advancing or delaying the main sleep period demonstrate that the WMZ is a robust phenomenon, which maintains its position independent of the timing of previous sleep. It depends solely on the circadian phase of process R. Thus, the only condition that might predict a diminution of the WMZ would be an amplitude reduction of sleep drive R^58,59^.

This is at least supported by observations in a study about age related changes in homeostatic and circadian regulation of sleep^68,69^. The authors reported that “the circadian regulation of sleep during a 40 h nap protocol was changed such that the circadian arousal signal in the evening was weaker in the older participants; more sleep occurred during the WMZ, and subjective sleepiness ratings in late afternoon and evening were higher than in younger participants”^69^. In addition, they found a diminished melatonin secretion and a reduced circadian modulation of REM sleep for the older participants. Both results were interpreted as evidence for reduced circadian influence. Furthermore they explicitly noted that, despite the fact that the older participants in the study had impaired sleep consolidation and reduced SWA levels, the relative SWA response to both high (sleep deprivation protocol) and low (nap protocol) sleep pressure conditions was similar to that of younger participants. They hence concluded that age related changes in sleep were due to a weaker circadian regulation rather than to a reduced homeostatic influence.

### The forced desynchrony experiment with severe sleep restriction

With reference to the above mentioned FD protocol with sleep restriction^43,44^, which poses problems to the 2-process model, we have reconstructed their published data within the framework of the S.R model^45^. In short, for this purpose we used as anchor points the experimental data from 12 circadian entrained, healthy males (age 26.8 ± 3.2 yrs) without sleep restriction (“Berlin”^55,59^) and the published data from the similarly characterized group of 14 males (age 21.8 ± 3.8 yrs) from the severely sleep restricted FD experiment^44^. We assumed that the original S and R curves from the Berlin sleepers optimally represent the adaptation part from the protocol of the FD experiment, with a sleep-wake ratio of 1:2 and bedtime from midnight to 8 am. Using these data as a starting point, we then estimated the further time course of S over the FD experiment by allowing S to decrease during sleep and increase during wakefulness, as determined by the sleep and wake times from the FD protocol^44^, their Fig. 1. while assuming the usual saturating exponential functions of S for increase and decrease^15,59^. To estimate the further time course of R, we fitted the original Berlin R-curve to the empirical REM latency data as given in Paech et al.^44^, their Fig. 2. which showed a remarkable, strongly attenuated circadian modulation with 1:5, but not with the habitual 1:2 sleep-wake ratio. In doing so, we assumed that the fitted R curve represents the final stage of an apparently attenuating effect that culminates toward the end of the FD experiment, at FD7. Interpolation between FD0 and FD7 was assumed to be the best reasonable estimate of the intermediate stages of this R-attenuation.

**Fig. 5:**
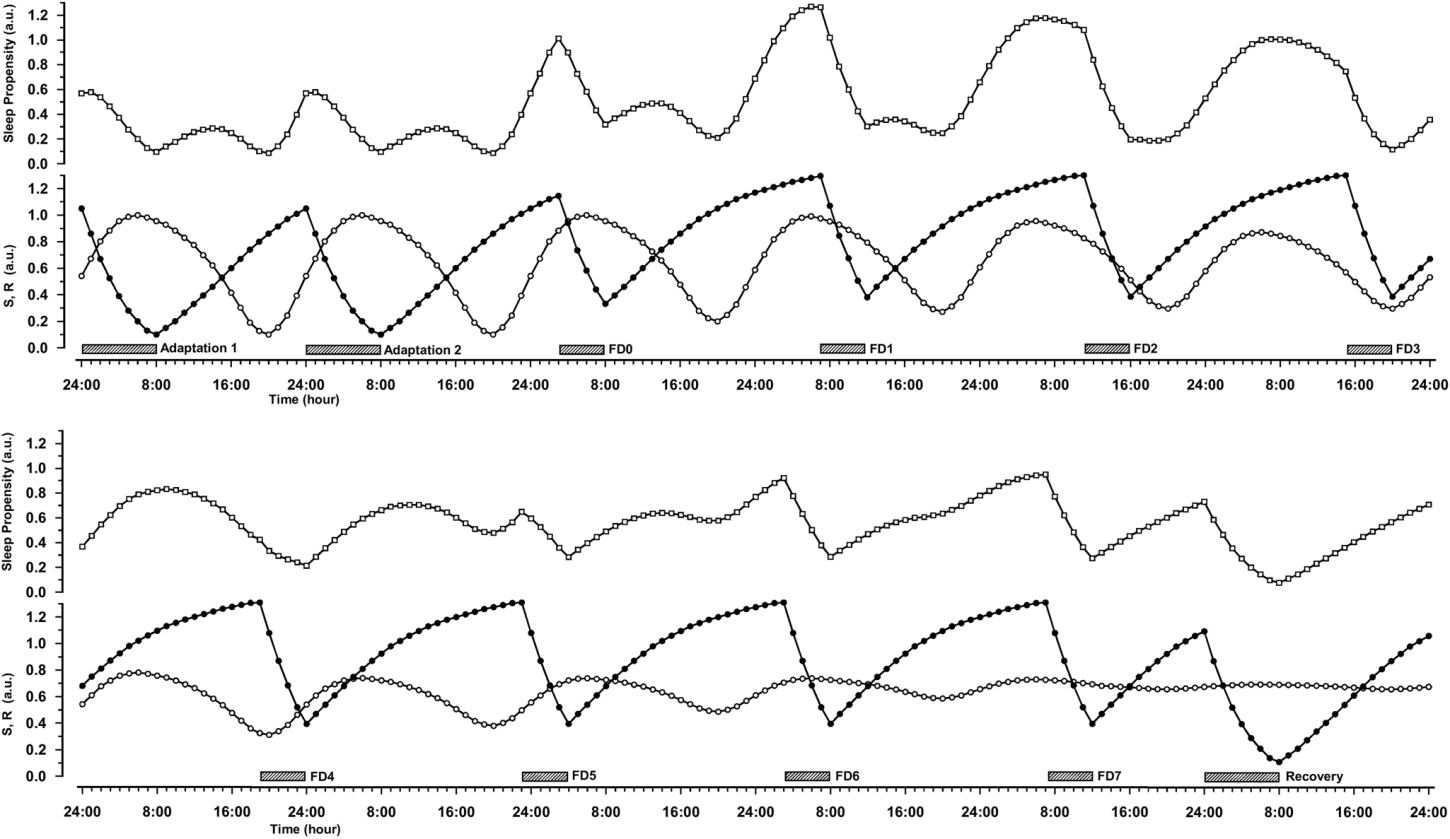
Simulated sleep propensity across the sleep restricted FD experiment. It covers the entire experimental time series^43,44^, including adaptation and recovery nights, and represents sleep propensity, together with the underlying processes S and R, as continuous time courses. The lower panel is the continuation of the upper one. Symbols are as indicated in Fig. 2. The hatched bars indicate the sleep opportunities as mentioned in ref. ^44^ (their Fig. 1). The various effects of the experiment are described in the text.

The result is shown in Fig. 5. The depicted time course over the entire experimental time frame reveals the dynamics of a number of phenomena in remarkable detail, characterized by roughly three phases. The first includes the adaptation section with transition to the FD protocol until FD1, and shows the 24-h bimodal pattern of two peaks and two minima in sleep propensity, similar to Fig. 2, but with increasing levels toward the end of this phase. The second phase, from FD1 to FD4, exhibits a highly pronounced unimodal 24-h pattern with three successively broadening peaks of extremely high sleep propensity. The high levels are clearly due to the prolonged episodes of accumulating S, running more or less in phase with the peaks of R, which greatly enhances their interaction. The ANZ disappears at FD2. Note that because the WMZs merge with the minima of S at the end of the sleep episodes, they can no longer be recognized as such in the sleep propensity time course. In the third phase, from FD5 to the recovery night, sleep propensity is mostly higher than baseline values and is eventually dominated exclusively by S, while the ever-increasing attenuation effect reduces the circadian amplitude of R, wiping out the WMZ.

An important question is why the circadian modulation of REM sleep propensity would become less pronounced in the course of the experiment. This may be due to the peculiar homeostatic response of REM sleep to deprivation, as described in animal experiments^56^, showing that dependent on circadian phase, REM sleep responds differently to deprivation. This also has been shown in an elegant experiment with the crepuscular (twilight-active) mammal *Octodon degus*, in which diurnal and nocturnal REM sleep deprivations provoked equivalent amounts of REM sleep debts, but a consistent REM sleep rebound was found only after nocturnal deprivation^70^. Although some caution is needed when mixing evidence from (polyphasic) animal studies with that from human studies, it is nevertheless not unlikely that the different times of REM deprivation, as a direct consequence of the sleep restricted FD protocol, cause varying effects on REM rebound, leading to a dampening of its circadian propensity profile.

Although the latter part of the discussion above is beyond the scope of this article, it shows that the S.R model allows a detailed description and discussion of the relevant processes, revealing a crucial role of the drive for REM sleep and its continuous interaction with S, that may explain the experimental effects. In contrast, the S-C model cannot explain the effects of the sleep-restricted FD experiment, because the normal course of core body temperature indicates an unchanged circadian modulation of C, and REM sleep has no relevance in the S-C model.

### Narcolepsy

**Fig. 6:**
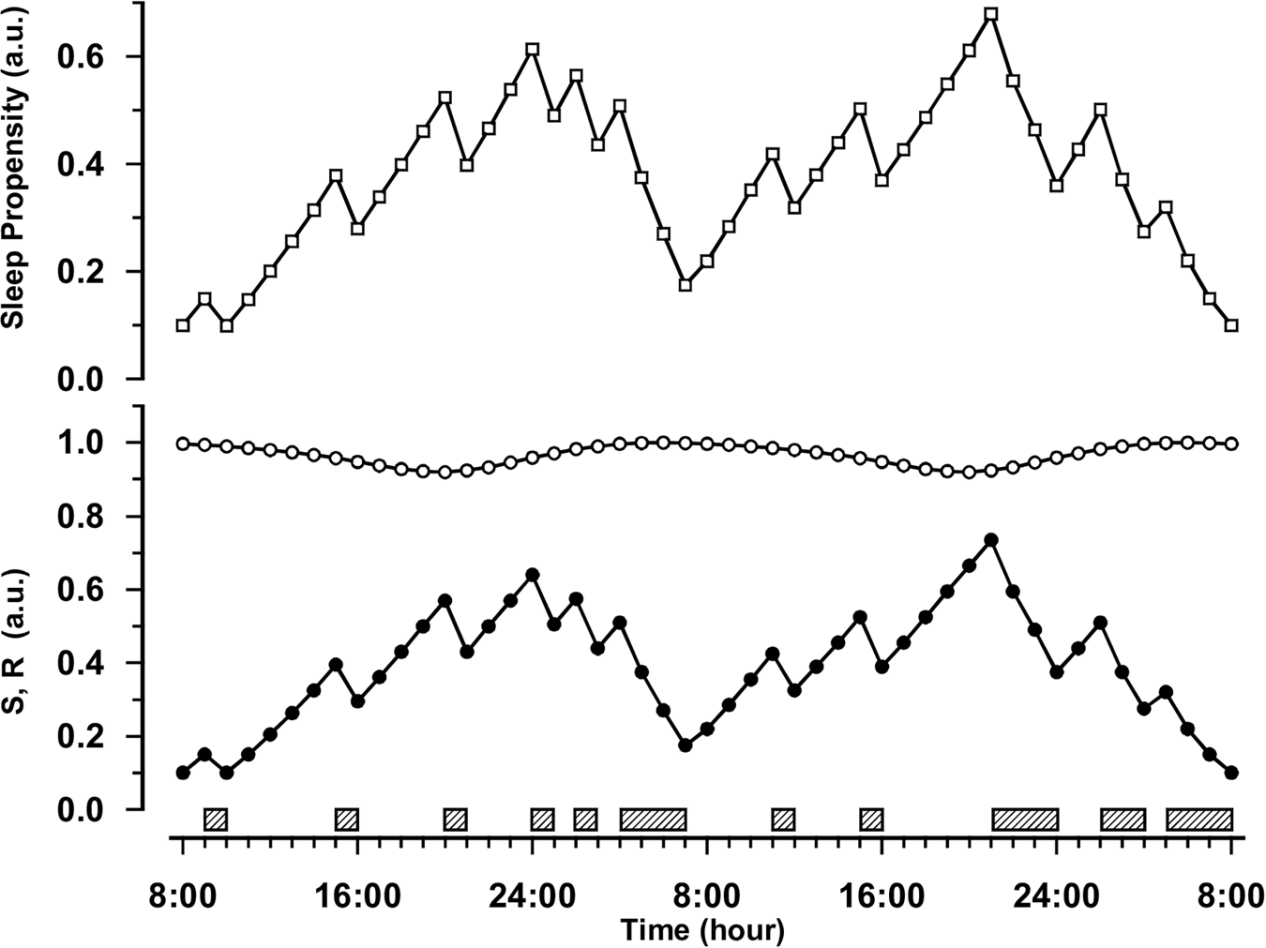
Simulated sleep propensity over 48 h of a hypothetical patient with narcolepsy. To obtain R, the original empirical R curve^55,59^ has been fitted to the combined REM-latency data from MSLTs of 31 patients with narcolepsy. Actometrically obtained sleep-wake times of a typical patient with narcolepsy have been used to illustrate a possible time course of S, under the assumption that homeostasis is functional in narcolepsy and thus the usual saturating exponential functions of S for increase and decrease can be used^15,59^. Symbols as indicated in Fig. 2. The hatched bars indicate sleep episodes.

Narcolepsy is a hypersomnia characterized by excessive daytime sleepiness, disrupted night sleep and abnormal manifestations of REM sleep, including cataplexy, sleep paralysis and hypnagogic hallucinations. The S.R model enables estimation of the time course of sleep propensity in narcolepsy because it implements sleep-onset-REMs, one of the diagnostic benchmarks of this pathology, as an obvious causal effect. In the S.R model, the extremely shortened REM latencies in narcolepsy indicate a severely elevated pressure for REM sleep and correspondingly extreme R levels, with greatly reduced circadian amplitude because of a ceiling effect (Fig. 6). This implies that there is no noticeable WMZ and that variations in the 24-h sleep propensity profile will be primarily dominated by variations in S. It has been reported in the literature that individuals with narcolepsy failed to have a decrease in sleep propensity late in the biological day^71^ and also, that spontaneous naps were commonly observed during this period^72^.

Assuming that sleep homeostasis is functional in narcolepsy-cataplexy^71,73^, the accumulation of S during wakefulness, multiplied by extremely high R values, will lead to steep increases in sleep propensity, ultimately resulting in irresistible naps. The low sleep efficiency at night, typical of narcolepsy, is explained by the fact that because of daytime napping, S never saturates to the usual high evening levels of healthy people under habitual, entrained circumstances. Rather, S remains at moderate levels that take little time to dissipate during nocturnal sleep, leading to short sleep episodes with intermittent episodes of wakefulness, and moderate or low sleep propensity (implying decreased sleep efficiency).

Although the S-C model would describe multiple napping and low nocturnal sleep efficiency of narcolepsy patients by reducing the distance between the upper and lower thresholds of process C, it fails to implement the significance of the typical sleep-onset-REMs, as REM sleep has no specific relevance in the model. Furthermore, circadian variation in core body temperature is reported to be normal in narcolepsy^71,74,75^, indicating an intact circadian timing system and thus a normal circadian modulation of process C.

## Discussion

In the above has been argued that a conceptual change of perspective on the two-process model could have implications that are very interesting to be explored further. Instead of having a homeostatic sleep process that is gated by a circadian alerting signal, the alternative view considers the interaction of two fundamentally different sleep-wake processes that are more or less equivalent in strength. When adopting a probabilistic approach, we obtain a logical argument for being allowed to describe the interaction of these two homeostatic and circadian sleep-wake processes by means of multiplication. As a result, thresholds and gates are no longer needed, but realistic and experimentally testable predictions about the temporal course of sleep propensity can still be made. Furthermore, because the circadian and homeostatic components are both considered genuine sleep-wake processes, REM sleep can be implemented as a key circadian component and hence this interpretation of the two-process model covers the “three primary states of being”^46,48–50^. Following the principle of Occam’s razor, the alternative conceptual interpretation could be preferable to the currently prevailing one, because it makes fewer assumptions with even broader theoretical coverage. One could argue that it gives the model additional explanatory capacity.

With the considered conceptual transition, the two-process model would turn away from the currently popular view of opposing homeostatic and circadian interaction (i.e. sleep versus wake). The probabilistic perspective requires that only the propensities for similar drives of the two sleep-wake processes can be multiplied: homeostatic sleep with circadian sleep (or, also possible, homeostatic wake with circadian wake, being the negative functions, or mirror images, of S and R). This fundamentally changes the dynamics of the interaction. A consequence of multiplication is that sleep propensity will be low when only one of the two constituent drives is low. Thus, the two processes are no longer intrinsically opposite, but operate in synergy at times when the drives tend toward similar extremes (both either low or high), and in opposition at times when the drives tend towards opposite extremes (one low and one high). Since the interaction is continuous, the immediate effects over time move between these two modalities (synergy versus opposition). This could have interesting implications, as the use of the two-process model has been gradually expanded over decades to include predictions on daily human sleepiness, alertness and neurobehavioral performance. Multiplying circadian and homeostatic wake propensity to obtain total wake propensity could imply that wake propensity due to low S (low levels of adenosine), for example in the morning after getting up, has a quality that is different from wake propensity due to low R (low levels of MCH, high levels of orexin), for example during the WMZ. With this line of reasoning a strong (negative) correlation between alertness and sleep propensity is supposed. However, these should not be assumed as simple antonyms. Alertness is more strongly dependent on task demands, situational factors, and the behavioral repertoire of a person than sleep propensity. The measurement of alertness differs substantially from that of sleep propensity, and thus, in such a context, one has to acknowledge methodological differences between the 2 concepts^59^.

It should be emphasized that process R in the S.R model is different from the process that Borbély originally presented as R in the extended two-process model^1^, ^p.200^, his Fig. 6. He assumed a constant level for R and defined its propensity as the difference between this level and the circadian wake propensity. Nevertheless, and this is remarkable, he indicated that in the extended model, REM sleep propensity “(…) corresponds to the circadian component of total sleep propensity (Process C)”^1^, ^p.200^ but unfortunately he never elaborated this further. Be that as it may, it would be consistent with considering process R in the S.R model as the key circadian sleep component and thus, also, a mirror image of the “putative circadian alerting signal”^42^, that was originally proposed by Dijk and Czeisler to explain the consolidation of nocturnal sleep and daytime wakefulness^20^. So far, however, details on how this consolidation could be achieved in the S-C model have not been published. The S.R model can adequately explain the mechanism behind this consolidation: the WMZ through the multiplication of high S values with very low R values, and the morning sleep consolidation through values of S that, although decreasing, are still high enough to be amplified by the then prevailing high R values^59^.

While it is assumed that the circadian influence from the central pacemaker on sleep propensity is mediated by R, the question arises whether circadian effects on sleep propensity are not nevertheless caused by direct influence of the circadian timing system itself (e.g. the SCN), in which case the circadian variation in R would be reduced to an epiphenomenon. However, situations with a demonstrably functioning circadian timing system, but in which R is hindered or prevented from exhibiting circadian modulation, indicate that this scenario is unlikely, though. Thus, the sleep restricted FD experiment^43,44^ demonstrated that, under prolonged conditions of 1:5 sleep-wake ratio, REM latency hardly showed circadian modulation, a WMZ could not be observed, and sleep propensity was very high at all circadian phases (Fig. 5, FD6 and FD7), although fluctuations in core body temperature, an established marker of the output of the human circadian pacemaker, suggested normal endogenous circadian cycles. A similar indication can be found in narcolepsy, where REM latency is strongly reduced day and night, causing R to exhibit extremely high levels that hardly vary over 24 h (Fig. 6). But circadian variation in core body temperature is reported to be normal in narcolepsy, indicating an intact circadian timing system^71,74,75^. Both examples make it very likely that it is indeed the dynamics of R which is responsible for the observed sleep propensity effects and not the circadian timing system per se.

The crucial role of orexin in the symptoms of narcolepsy-cataplexy became clear when the neuropeptide was found to be absent in patients with this pathology^76^. Its significance for the circuitry of REM sleep generation, together with its ‘natural reciprocal’, melanin concentrating hormone (MCH), was recognized in the subsequent decades^77,78^. The idea that one of orexin’s primary roles is to promote wakefulness became particularly widespread, not least because of the promising development of orexin receptor antagonists for the treatment of insomnia^79^. Nevertheless, it is emphasized here that orexin, as part of the orexin/MCH system, should be regarded as a neurotransmitter that inhibits REM sleep rather than as a wakefulness promoter in itself^80^. Thus, it is appealing to hypothesize that the circadian profile of the orexin/MCH system could represent a neurophysiologic correlate of process R, analogous to how the homeostatic profile of the adenosine system, at least with prolonged wakefulness^81^, could be considered a neurophysiologic correlate of process S.

The strength of the two-process model is the simplicity with which it combines the two fundamental processes believed to regulate the sleep-wake cycle. With the considered shift in conceptual perspective, the two-process model would even gain in simplicity, as thresholds and gating become unnecessary, but would nevertheless also gain in explanatory power, as it encompasses the two main sleep drives SWS and REM, which through multiplication allow experimentally verifiable quantification of sleep propensity. Because the model under the alternative perspective assumes mathematically the same fundamental constituent processes (circadian and homeostatic), it will not conflict with the vast majority of experimental research published under the perspective of the S-C model. Moreover, most of those studies were not designed to specifically test the putative threshold nature of the interaction. However, a shift in perspective on the two-process model, as discussed in this treatise, may enable new conceptual interpretations of previous results and promote new insights for experiments that can further enrich the field of sleep research. The scientific community is called upon to further explore this interesting, original idea, once also initiated by Alexander Borbély.

## Data Availability

Data of this study are available from the author upon reasonable request.
